# A low-cost, autonomous mobile platform for limnological investigations, supported by high-resolution mesoscale airborne imagery

**DOI:** 10.1371/journal.pone.0210562

**Published:** 2019-02-14

**Authors:** D. Andrew Barry, Jean-Luc Liardon, Philippe Paccaud, Pascal Klaus, Nawaaz S. Gujja Shaik, Abolfazl Irani Rahaghi, Ludovic Zulliger, Jérôme Béguin, Beat Geissmann, Stepan Tulyakov, Anton Ivanov, Htet Kyi Wynn, Ulrich Lemmin

**Affiliations:** 1 Laboratoire de technologie écologique (ECOL), Institut d’ingénierie de l’environnement (IIE), Faculté de l’environnement naturel, architectural et construit (ENAC), Station 2, Ecole polytechnique fédérale de Lausanne (EPFL), Switzerland; 2 Space Engineering Center (ESC), Station 13, Ecole polytechnique fédérale de Lausanne (EPFL), Switzerland; University of Electronic Science and Technology of China, CHINA

## Abstract

Two complementary measurement systems—built upon an autonomous floating craft and a tethered balloon—for lake research and monitoring are presented. The autonomous vehicle was assembled on a catamaran for stability, and is capable of handling a variety of instrumentation for in situ and near-surface measurements. The catamaran hulls, each equipped with a small electric motor, support rigid decks for arranging equipment. An electric generator provides full autonomy for about 8 h. The modular power supply and instrumentation data management systems are housed in two boxes, which enable rapid setup. Due to legal restrictions in Switzerland (where the craft is routinely used), the platform must be observed from an accompanying boat while in operation. Nevertheless, the control system permits fully autonomous operation, with motion controlled by speed settings and waypoints, as well as obstacle detection. On-board instrumentation is connected to a central hub for data storage, with real-time monitoring of measurements from the accompanying boat. Measurements from the floating platform are complemented by mesoscale imaging from an instrument package attached to a He-filled balloon. The aerial package records thermal and RGB imagery, and transmits it in real-time to a ground station. The balloon can be tethered to the autonomous catamaran or to the accompanying boat. Missions can be modified according to imagery and/or catamaran measurements. Illustrative results showing the surface thermal variations of Lake Geneva demonstrate the versatility of the combined floating platform/balloon imagery system setup for limnological investigations.

## Introduction

Worldwide, the quality and quantity of inland waters is a preoccupation for water resource management (e.g., [1–4]). Our focus here is on lakes, as a core part of the inland water cycle. The threats to ecosystem services provided by such resources are unlikely to be diminished in the foreseeable future (e.g., [5–9]). On the contrary, increasing population and associated demands, for instance, for agricultural products, will continue to underscore the value of water resources. At the same time, the on-going impacts of climate change will modify precipitation patterns and timing, and hence variations in water availability (e.g., [10–12]). Such impacts require quantification based on strategic environmental monitoring and assessment (e.g., [13]). For example, the need for clear information that can guide hydro-climate monitoring development was investigated using data from 14 major hydrological basins across the pan-Arctic [14]. Another impact of climate change is the increase in lake temperature observed over widespread areas [15–17]. The magnitude of these changes varies within and between lakes [18]. Some studies proposed methods to prioritize monitoring of future climate change hot spots of surface waters [19] as well as strategies for spatially sampling water bodies [20]. In this context, and more generally to ascertain spatial and temporal variability of lake waters, accurate data gathered at appropriate intervals are essential.

The task of characterizing lake waters is assisted by the many recent technological developments in instrumentation. Specifically, lowering cost barriers without significantly compromising sensor precision makes widespread data gathering feasible. Combined multidisciplinary research and education endeavors, such as Project EDDIE (Environmental Data-Driven Inquiry and Exploration), which relies on a variety of different environmental datasets, emphasize the value and also global interests of such widespread, multivariable data. For lakes, GLEON (Global Lake Ecological Observatory Network), which is motivated by researchers having the goal to share and interpret “high resolution sensor data to understand, predict and communicate the role and response of lakes in a changing global environment,” is one of the leading worldwide limnological networks for such data.

Traditionally, limnological monitoring, either for routine or for research purposes, relies on sparsely spaced point measurements (e.g., [21, 22]), or 1D profiles (partially) spanning the water column (e.g., [23–25]). However, imagery from satellites, aircraft or drones also provides valuable information on lake waters (e.g., [26–32]), although data are limited to the surface or near-surface layer.

Along with the trend in reducing sensor costs, the availability of open-source software as well as technical innovations and production improvements make feasible the creation of autonomous, mobile platforms for data gathering without high equipment or personnel costs. For lakes, several autonomous or semi-autonomous platforms, i.e., unmanned and/or autonomous surface vehicles (USV/ASV, all acronyms are listed in Table 1), are described in the literature [33]. Most were developed for a single type of mission (e.g., bathymetry [34] or surface microlayer analysis [35]). These platforms involve customized designs that are relatively expensive and require substantial effort to modify, for instance to incorporate new sensors. Indeed, publications describing such craft tend to emphasize control and navigation solutions instead of modular sensor management [36].

**Table 1:** Acronyms.

Acronym	Definition
ADC	Analog-Digital Converter
ADCP	Acoustic Doppler Current Profiler
AHRS	Attitude and Heading Reference System
ASV	Autonomous Surface Vehicle
AVHRR	Advanced Very High Resolution Radiometer
BBB	BeagleBone Black
BLIMP	Balloon Launched Imaging and Monitoring Platform
COTS	Commercial Off-The-Shelf
CPU	Central Processing Unit
CSI	Camera Serial Interface
CSV	Comma-Separated Values
CTD	Conductivity, Temperature, Depth
DC	Direct Current
DOF	Degrees Of Freedom
EDDIE	Environmental Data-Driven Inquiry and Exploration
FOV	Field Of View
FSK	Frequency-Shift Keying
FTP	File Transfer Protocol
GLEON	Global Lake Ecological Observatory Network
GPS	Global Positioning System
I^2^C	Inter-Integrated Circuit
ImPROV	Imaging Package for Remotely Operated Vehicle
IMU	Inertial Measurement Unit
Li-Po	Lithium-Polymer
LSWT	Lake Surface Water Temperature
LTE (4G)	Long-Term Evolution
LWIR	Long-Wavelength Infrared
MEMS	Micro-Electro-Mechanical System
MLESAC	Maximum Likelihood Estimation Sample Consensus
MSER	Maximally Stable Extremal Regions
PCB	Printed Circuit Board
PWM	Pulse Width Modulation
RC	Radio Control
RGB	Red Green Blue
SD	Secure Digital
SPI	Serial Peripheral Interface
SSH	Secure Shell
SURF	Speeded-Up Robust Features
UART	Universal Asynchronous Receiver/Transmitter
UDP	User Datagram Protocol
USB	Universal Serial Bus
USV	Unmanned Surface Vehicle
VPN	Virtual Private Network
WLAN	Wireless Local Area Network

Our goal is to present two complementary platforms for limnological investigations. First, we describe a low-cost, autonomous mobile platform suitable for both research purposes and for routine measurements on lakes. Besides lowering the cost barrier, the design enables (i) the ability to record from a range of instruments, (ii) real-time data accessibility, (iii) real-time mission modifications and (iv) a modular equipment configuration to minimize deployment and post-mission retrieval time and effort. These features were realized using the catamaran-based craft described below. Although the craft is autonomous, legal restrictions in Switzerland (usage location) prevent fully remote deployments, i.e., the craft must be within sight of an accompanying boat. The second platform consists of a He-filled balloon equipped with an instrument package for surface imaging. Like the catamaran, our aerial imaging system is low-cost and easily deployed. Our focus for aerial imagery is the lake surface thermal structure, although other imagers could be used. The ground crew can identify areas of interest using the real-time thermal images, e.g., areas with cold-warm surface temperature patches or streak-like structures, and carry out simultaneous ZiviCat measurements in the identified areas. An example of such data is presented below in the “Mission Results” section. The combination of these two independent platforms offers the unique ability to carry out measurement campaigns that sample areas of the lake according to real-time aerial imaging information as well as measurements from the autonomous craft.

## Methodology

The measurement system is comprised of two independent systems: An autonomous floating mobile platform named ZiviCat, and the accompanying airborne instrument package referred to as BLIMP (Balloon Launched Imaging and Monitoring Platform). While independent, these systems can be monitored simultaneously and controlled using a base-station computer running an interface program for each platform, through which the initial parameters of the mission can be set and the measured data monitored in real time.

In the following subsections, the systems of both platforms are described in the following order:

- ZiviCat: Autonomous floating mobile measurement platform
  - ZiviCat Hardware
  - ZiviCat Software
  - ZiviCat Base Station
  - ZiviCat Performance Summary
- BLIMP: Balloon Launched Imaging and Monitoring Platform
  - BLIMP Hardware
  - BLIMP Software
  - BLIMP Base Station

Detailed information for each platform is provided in the Supporting Information (ZiviCat in Part A of S1 Text and BLIMP in Part B) of S1 Text, with an overview presented below. The Supporting Information includes hardware descriptions and web links.

### ZiviCat: Autonomous floating mobile measurement platform

ZiviCat is an autonomous surface vehicle (ASV) used for limnological research. The catamaran’s twin-hull layout is ideal for stability and in situ measurement setups. The associated control software was designed to permit adoption of different sensors. The overall platform is built on modular components, and can accommodate or be adapted to different sensors.

#### ZiviCat hardware

**Fig 1:**
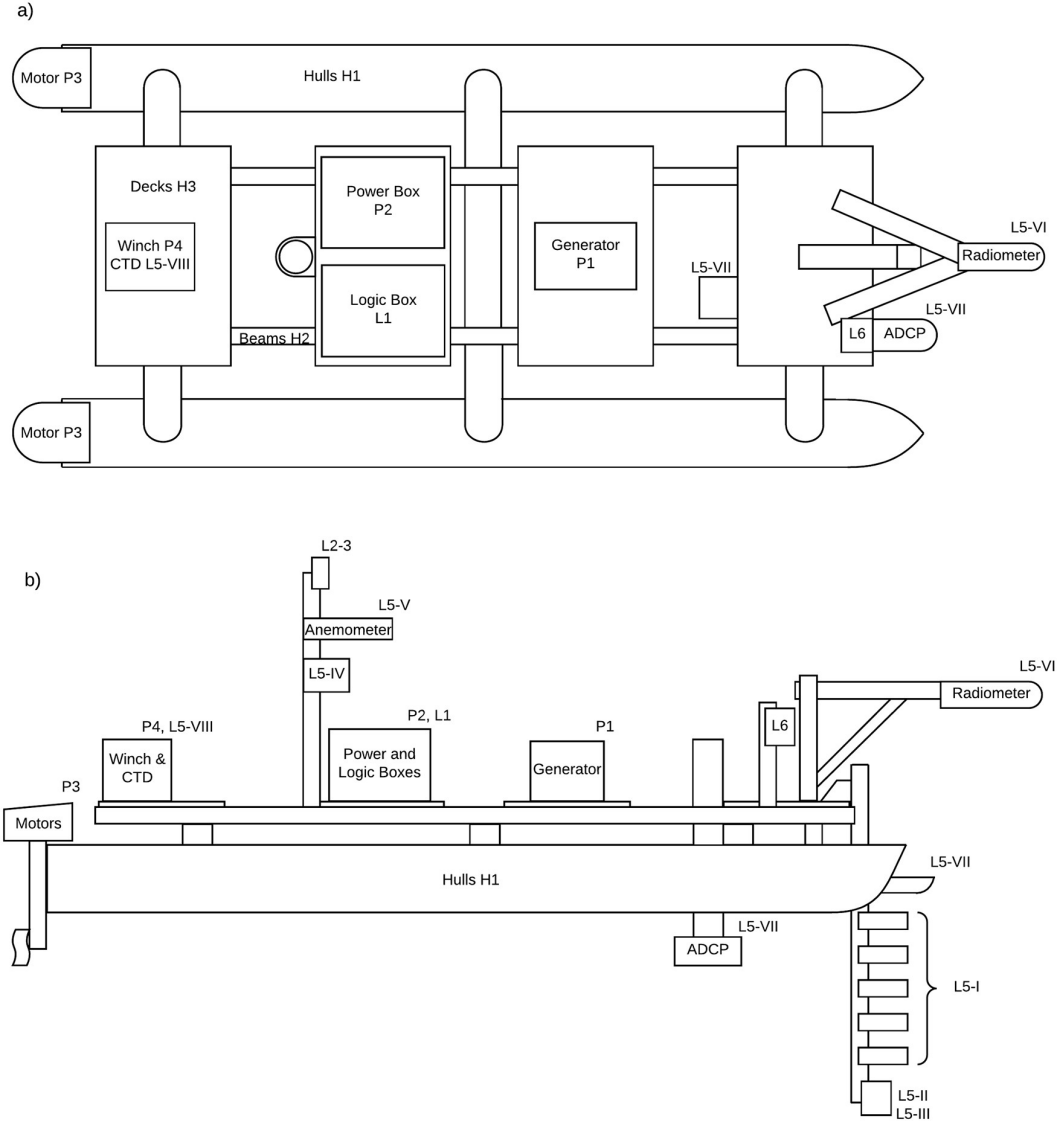
ZiviCat platform schematic. The modified Hobie Cat 17 is configured with separated decks for equipment placement. The large gap in the middle of the craft, which is for deploying equipment between the hulls, could be used for additional decking space, if needed. More details are provided in the text. a) Top view, b) Side view.

The ZiviCat is built on the frame of a commercially available catamaran (H1), i.e., Hobie Cat 17 (last accessed 17 November 2018) frame, which is 5.18-m long, 2.41-m wide (Fig 1 and Part A of S1 Text). Only the hulls and crossbars (joining the two hulls) were retained. Aluminum beam profiles (H2) were installed to support four water-resistant plywood sheets (H3), thereby creating separate decks for mounting equipment. As described below, modular power and control systems were created to minimize mission-preparation time.

**Fig 2:**
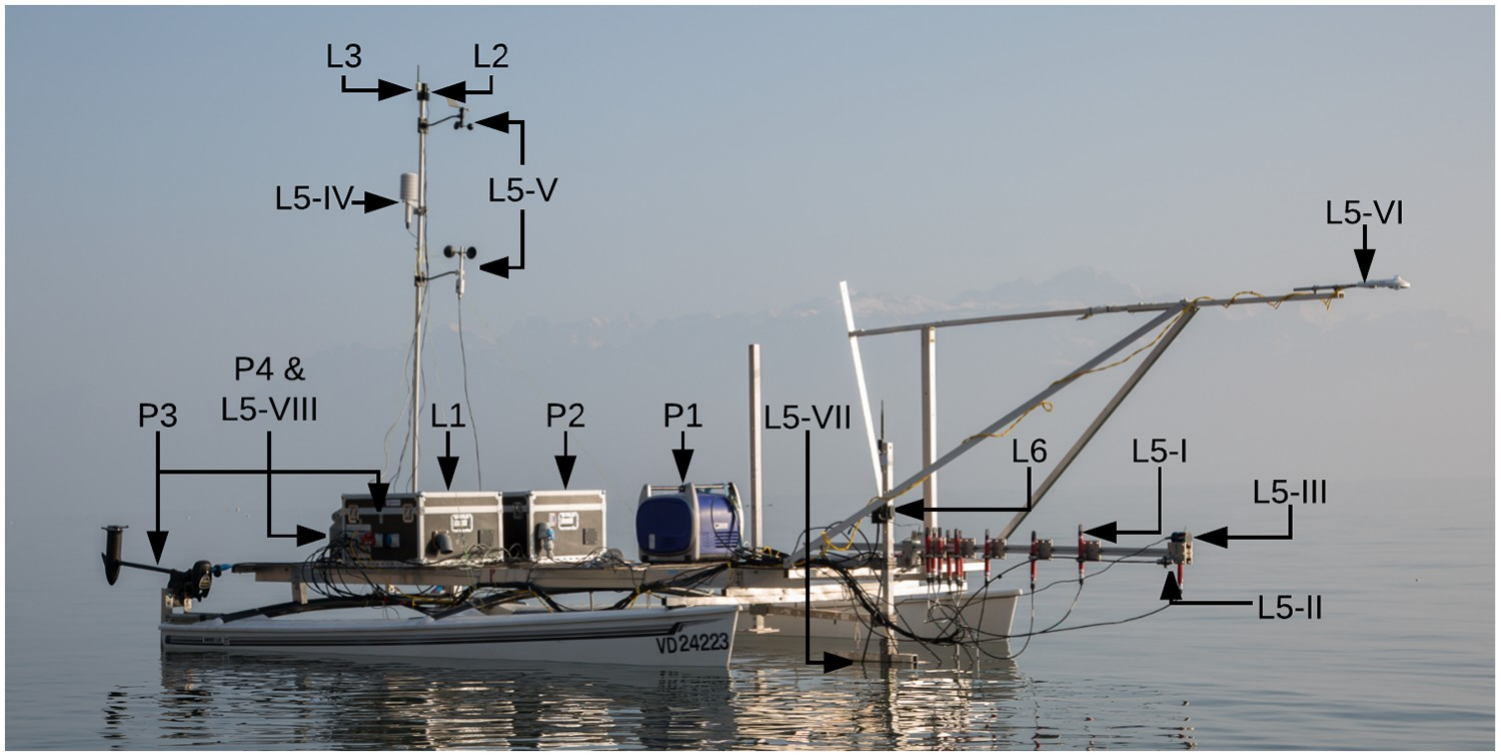
ZiviCat platform setup. The front sensor bar is fixed horizontally for shallow water navigation. The equipment numbering links to the hardware description list in Part A in S1 Text.

**Fig 3:**
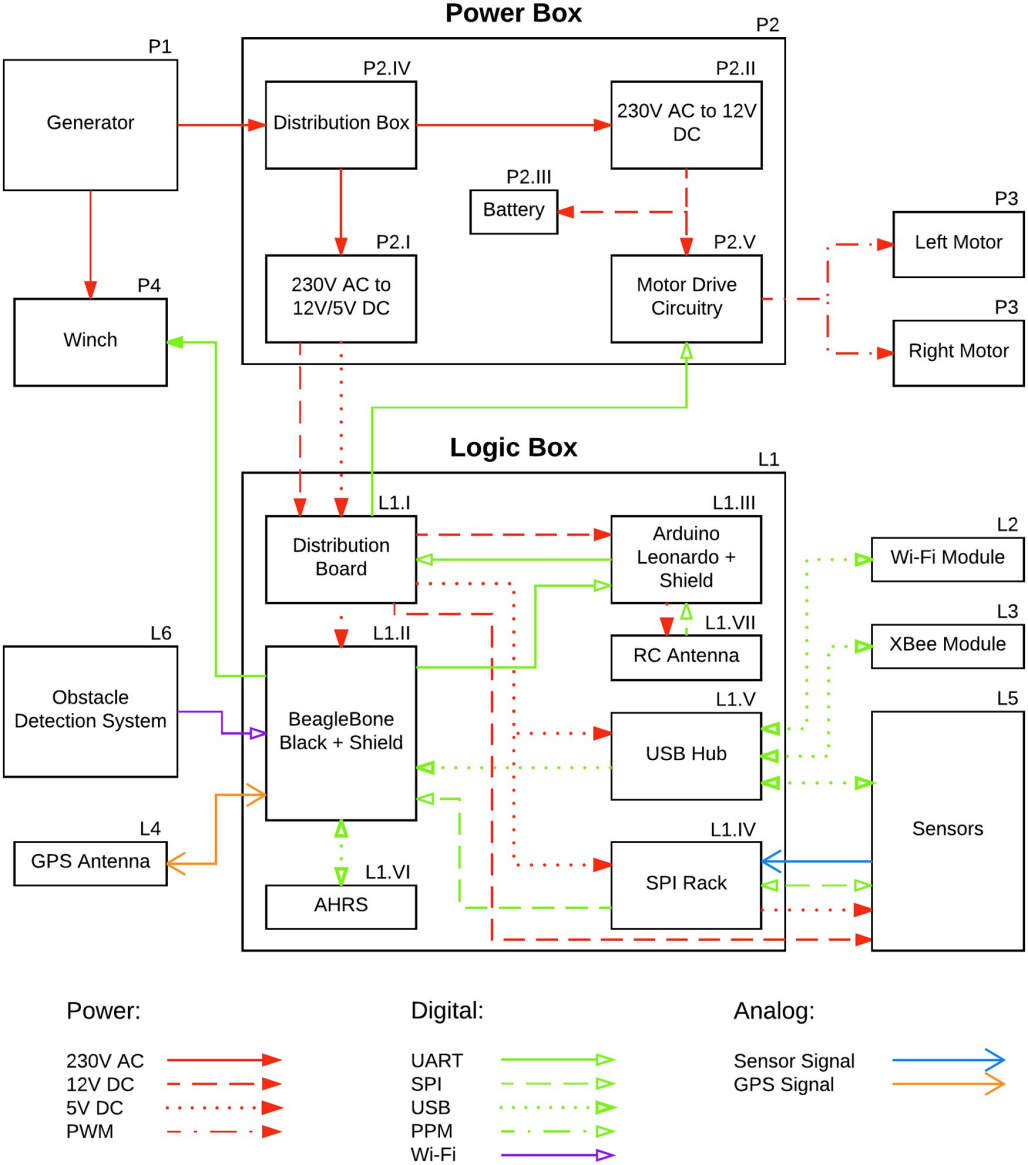
ZiviCat hardware system schematic. Relevant components and their connections are shown. The numbering refers to the hardware description list in Part A in S1 Text.

The decks in Fig 1 are used as follows (see Figs 2 and 3 for specific components):

- The aft deck supports a winch (P4) for lowering and raising a CTD profiler (L5-VIII).
- The center-aft deck holds the system electronics, separated into two boxes—power management (P2) and system control (L1)—for convenient transport and setup. A small mast holds the communication antennas (L2/L3), two anemometers (L5-V), and a humidity sensor (L5-IV). The power and logic systems are schematized in Fig 3. The power system encompasses a Yamaha EF2400Is generator (P1), a distribution box (P2-IV), two DC regulators (P2-I/II), a lead-acid 12V battery (P2-III), a motor control circuit (P2-V), and two Minn Kota Traxxis motors (P3). In the distribution box, at boot up, a power resistor is connected in series between the generator and the regulators, to limit the inrush current to the generator. After 1 s, a time-delay relay short-circuits the resistor. One regulator (P2-I) is used as a power supply for the logic system, the other (P2-II) is used to power the motors. The battery, mounted in parallel to the regulator for the motors, absorbs inrush and reverse currents, which are not managed by the regulator. The motor control circuit is a Sabertooth dual motor driver board.
- The center-front deck holds a gasoline generator (P1), which delivers all electrical power for the system.
- The forward deck supports a downward-pointing bar (Fig 2), upon which sensors are mounted such that their sampling region is unaffected by hull disturbances during forward motion. As presently configured, the board holds the following sensors: (i) 10 temperature sensors (L5-I), a pressure sensor (L5-II), and an echo sounder (L5-III) placed on a vertical, 2-m aluminum beam (hinged for navigation in shallow areas), which sample the top 1.5 m of the water column; (ii) a radiometer (L5-VI) mounted at the end of a horizontal mast for determining radiative energy flux; (iii) an obstacle detection system (L6); and (iv) two ADCPs (L5-VII).
- Two Minn Kota Traxxis motors (P3) at the back of the hulls are used for differential control (i.e., rudderless navigation) of the ASV. A total thrust of approximately 245 N allows a speed of about 2 m/s without payload, and 1 m/s when fully loaded with the setup described here. This speed difference is mainly due to the drag induced by the instruments taking measurements within the water profile. The fuel capacity permits 8-h missions before refueling is necessary.

The logic system in Fig 3 was developed to be easily implemented in any differential surface vehicle. It enables autonomous navigation, communication, and sensor recording/monitoring capabilities. The power system is independent and can be changed to suit a particular platform, by replacing the generator with Li-Po batteries for instance. The system is built around a lightweight Beaglebone Black (BBB, L1-II) Linux computer that runs software as described below. A custom shield is mounted on it allowing for simple connection of most peripherals. The ZiviCat has a GPS (L4) and an IMU (L1-VI) for navigation and heading information. Because many sensors are used, a versatile SPI-interfaced PCB rack (L1-IV) was created in-house using a 3D printer. Each sensor is connected to its own board on the rack, implementing at least a microcontroller to retrieve the sensor data and to communicate with the BBB through SPI. Templates for the boards are made as needed, e.g., a board based on an Arduino for modular capability. Some PCB designs can be used for different sensors (e.g., analog sensors or similar communication protocol). A multiplexer controlled by the BBB determines which board on the rack is addressed. The USB hub (L1-V) accommodates peripherals needing that connection type. The configuration includes two communication protocols (for redundancy), an XBee module (L3) and a Wi-Fi USB dongle (L2). Independent of the two protocols is an RC antenna (L1-VII) for manual control of the craft.

An Arduino Leonardo (L1-III) is a convenient solution for remote control capabilities. Its shield simplifies the connections of the peripherals and implements a multiplexer that controls the source of the commands to the motor driver. In autonomous mode, the BBB commands simply pass through the multiplexer. This mode is overridden by an operator via the remote control antenna (L1-VII), in which case the Arduino takes control of the driver and transmits commands to it.

Finally, for safe autonomous navigation, a camera-based obstacle-detection system (L6) was developed. Due to the legal limitation in Switzerland that mandates fully supervised deployments, this system is for demonstration purposes only. The system was developed using an inexpensive webcam with associated image processing routines carried out using a dedicated single-board computer [37]. Information on detected obstacles, their relative angular position and estimated distance are received by the BBB so that it can, for example, modify the catamaran’s trajectory accordingly, or simply shut down the motors. The system, which updates at around 4 Hz, connects to the ASV’s communication system, and sends frame-based obstacle(s) data through UDP to the operator.

#### ZiviCat software

**Fig 4:**
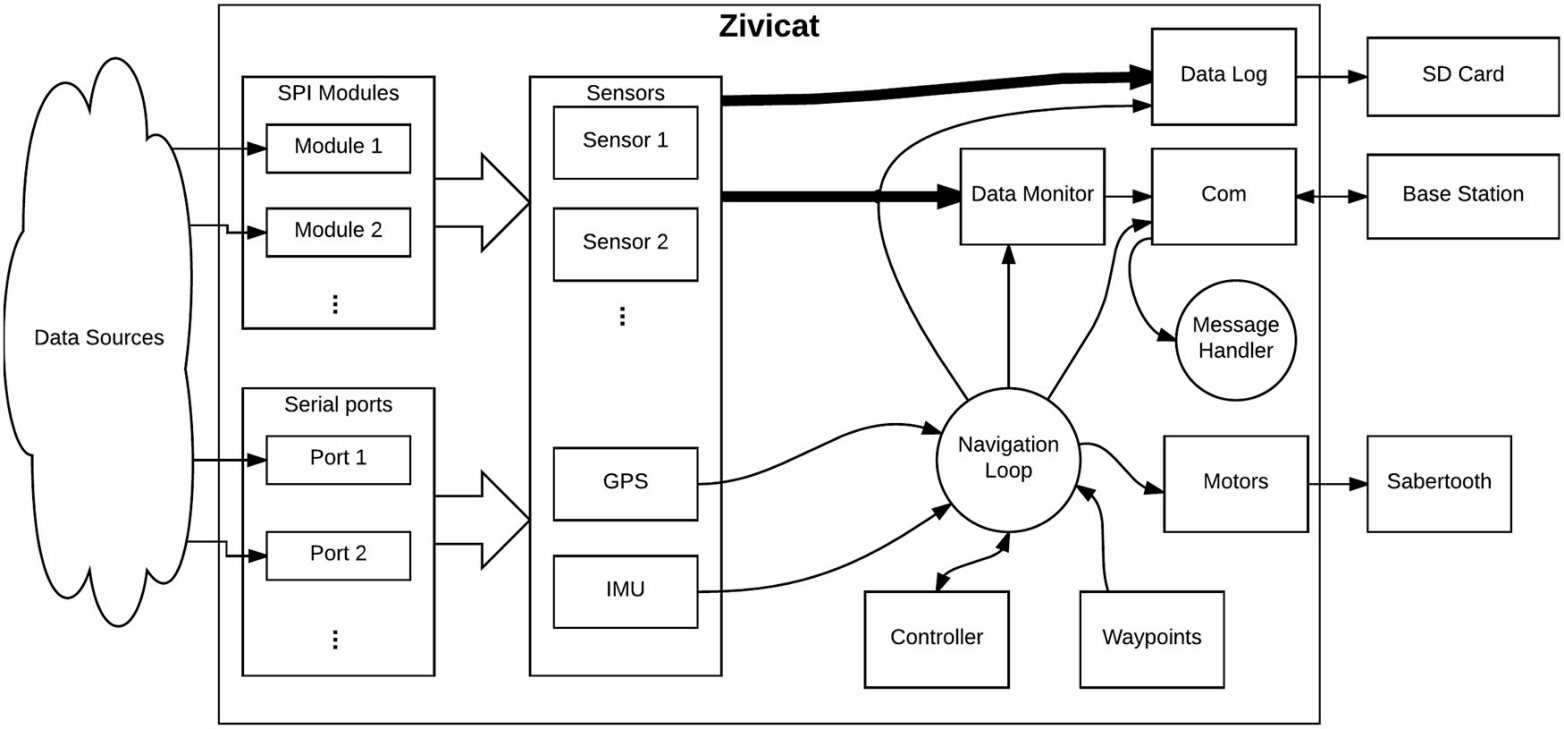
ZiviCat software schematic. The program is decomposed into multiple independent classes (described in the text), working with asynchronous events.

The C++ software architecture, shown in Fig 4, is organized in several modules running concurrently. The system uses asynchronous events, which operate in a similar manner to interruptions but are handled directly by the Linux Kernel instead of the program itself. This event-based structure allows all the modules to run independently without the need for multiple threads or processes, as well as communication between the different parts of the vehicle control system, running at different frequencies.

The main class, Zivicat, controls the whole program and all the modules (Fig 4). The other modules (i.e., subparts of the programs running independently from each other, not to be confused with classes) are the sensor modules, the acquisition SPI modules, the data log module, the variable monitor module, and the communication module. A sensor is represented by its own module and, if connected to the SPI rack, an additional SPI module. Sensors connected directly to the BBB use serial communication that is implemented inside the sensor module. The Com module communicates with the base station. It sends the received commands to the Zivicat module for execution through a message handler. The navigation is handled by a loop within the Zivicat module. The computed commands are sent to the motor driver through a motor control class. All the measured data are sent to the Data Log and Variable Monitor modules for record and display purposes, respectively. The different modules function as follows:

- The SPI module handles the SPI communication with the rack to retrieve raw sensor values. Each module represents a PCB on the SPI rack.
- The Sensor modules represent the physical sensors. Their data are obtained from either their assigned SPI module or from a serial connection on the BBB. A single sensor could use multiple acquisition sources, the same way a single SPI module could be used for multiple sensors.
- The Data Log module stores all the sensor and mission-critical data in an internal format. For export, the log file is parsed to a CSV table using the base station program.
- The Variable Monitor module handles the selection of data to be streamed to the base station (through the Com module). It receives instructions on which parameters from which sensors are to be streamed to the base station and transmits the requested information. The frequency of data sampling and streaming are set in the base station program (typically 1 Hz).
- The Com module handles all the communication with the base station. It does not process any data packets, but only routes them either to the Zivicat module or to the base station.
- The Zivicat module, running at 10 Hz, controls the whole program. It uses the GPS and IMU-based heading data it receives, along with the waypoint information from the base station, to compute the motor commands when in autonomous mode. It handles the speed of each motor, as well as possible emergency situations such as loss-of-signal with the base station. In such situations, the system is configured for three options: stop the mission, suspend the mission until the system is again fully functional (e.g., communication is re-established), or continue the planned mission. It can also create new SPI and sensor modules, if required by the base station, and controls their update frequencies.

#### Base station

**Fig 5:**
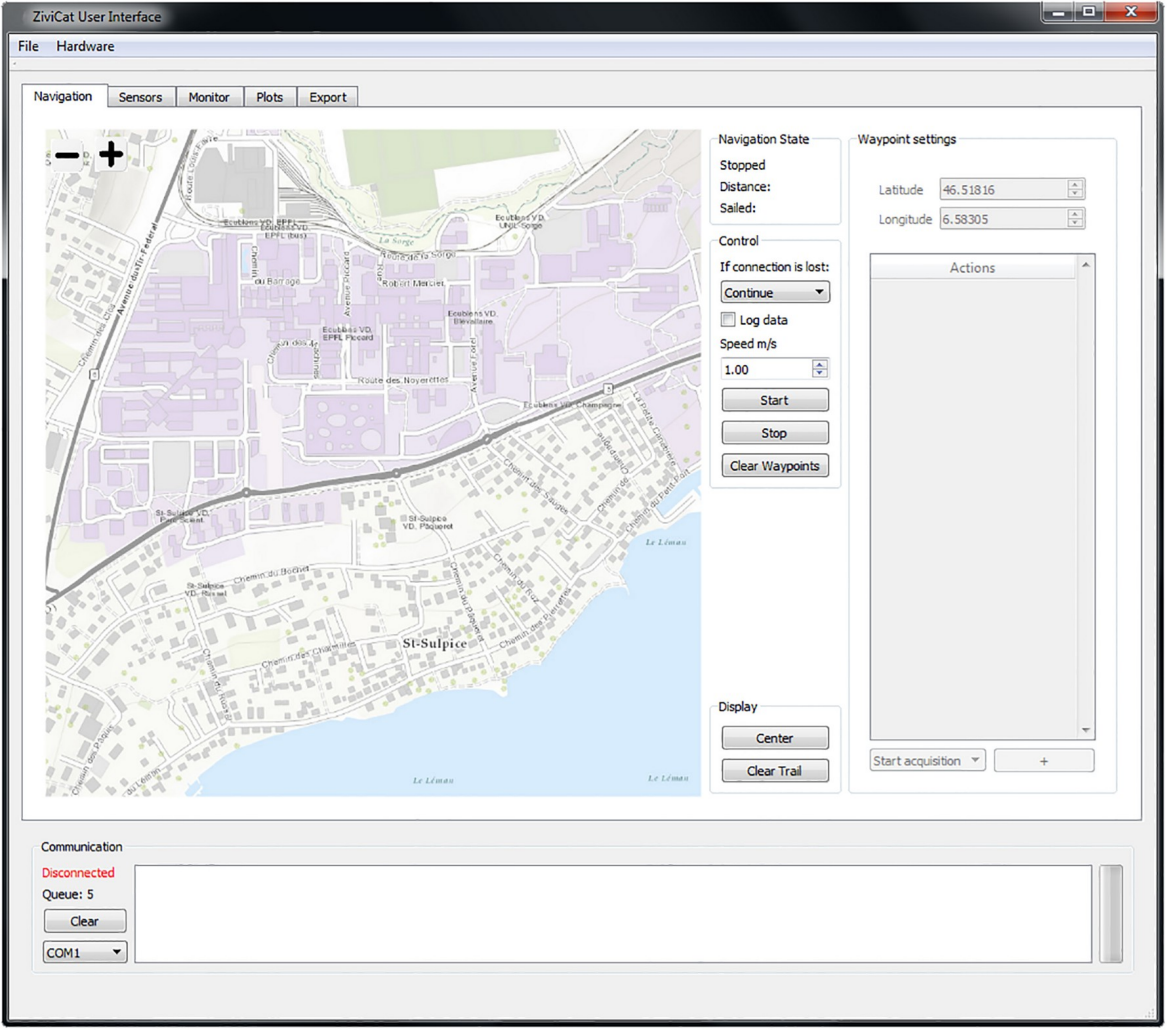
Screenshot of the user interface of the ZiviCat base station program. The operator selects different tabs as needed for mission setup, ZiviCat control and monitoring of measurements taken with the different sensors. The map (for illustrative purposes only) in the left panel is reprinted from USGS Earth Resources Observation and Science (EROS) Center.

The base station, a laptop running Windows 7 or later, communicates with the ZiviCat platform through an XBee antenna. The operator runs a program (Fig 5) that initializes the different sensors as required for the mission. It defines and loads mission parameters (such as waypoints, speed, update rate for each sensor, etc.), and defines emergency behavior (e.g., communication loss or obstacle detection). During a mission, the base station program plots real-time data from operator-selected sensors and, if needed, modifies the mission route. Note that we did not implement the “internet-over-4G” communication (described in Part C of S1 Text) used with the BLIMP (section BLIMP Software below) since in our deployments the craft must be kept within sight. However, the 4G communication could readily be implemented. This would enable mission planning, sensor monitoring and control of the craft at a given site (with mobile telephone network coverage) from any other site.

The BBB can also be accessed by Wi-Fi, as already described. Although Wi-Fi can be used during a mission, due to its short range it is principally used post-mission to access data with an FTP client such as WinSCP (last accessed 17 November 2018). Mission data, which are stored on an on-board SD card, can also be retrieved manually. Other tasks are also achieved via Wi-Fi, e.g., SSH can be used to reprogram the board, or to test program modifications such as communications with new sensors. Post-mission, the base station program parses the data into a standardized format for later analysis.

The base station is configured so that, at any time, the catamaran’s autonomous mode can be overridden for manual motor and navigation control using an RC transmitter. This is particularly useful in harbors or other circumstances where direct operator control of the craft is required for practical or legal reasons.

#### ZiviCat performance summary

Table 2 shows a comparison between ZiviCat and a recent autonomous craft, the HydroNet [38]. The table shows that the different craft have an overlapping range of capabilities. The HydroNet craft is configured to sample water for quality monitoring at up to 50-m depth, whereas ZiviCat, in its current configuration, can only take measurements at such depths using its CTD probe. Other major differences are the platform and the design details, which affect the cost and adaptability of the craft. The ZiviCat uses an existing commercial catamaran whereas the HydroNet is custom-built. In addition, ZiviCat uses existing COTS hardware, where feasible.

**Table 2:** Comparison of ZiviCat with a recently developed ASV.

	HydroNet [38]	ZiviCat
Hull(s)	Custom-made carbon fiber	Hobie Cat 17
Localization (GPS)	Yes	Yes
Sensors	Chemical sensors (Hg, Cr, Cd), plus optical detection of oil slicks	Anemometer, temperature profiles, air temperature, CTD, downward and upward pointing ADCPs, echo sounder, radiometer, water pressure, relative humidity
Dimensions	1.91 m length and 1.164 m width	5.6 m length and 2.5 m width
Communication	433 MHz using the AmI protocol	(i) Wi-Fi and (ii) XBee (to groundstation) plus manual override using a radio controller
Water Sampling	Yes, from specified depths up to 50 m	No
Power management	Yes	Yes
Obstacle avoidance	Yes	Yes
Power source	Li-Po batteries (1800 Wh total)	Petrol generator
Propulsion	Electric outboard motors (two)	Electric outboard motors (two)
Autonomous navigation	Yes	Yes
Estimated autonomy	6.5 h	8 h
Estimated maximum mission distance	26 km	28–56 km

### BLIMP: Balloon Launched Imaging and Monitoring Platform

The BLIMP is an aerial imaging system. It is used alongside the ZiviCat platform for high-resolution mesoscale thermal and RGB imaging of the lake surface. Post-mission, the images are stitched for a complete picture of the targeted area [39]. The system is described in the following sections.

#### BLIMP hardware

**Fig 6:**
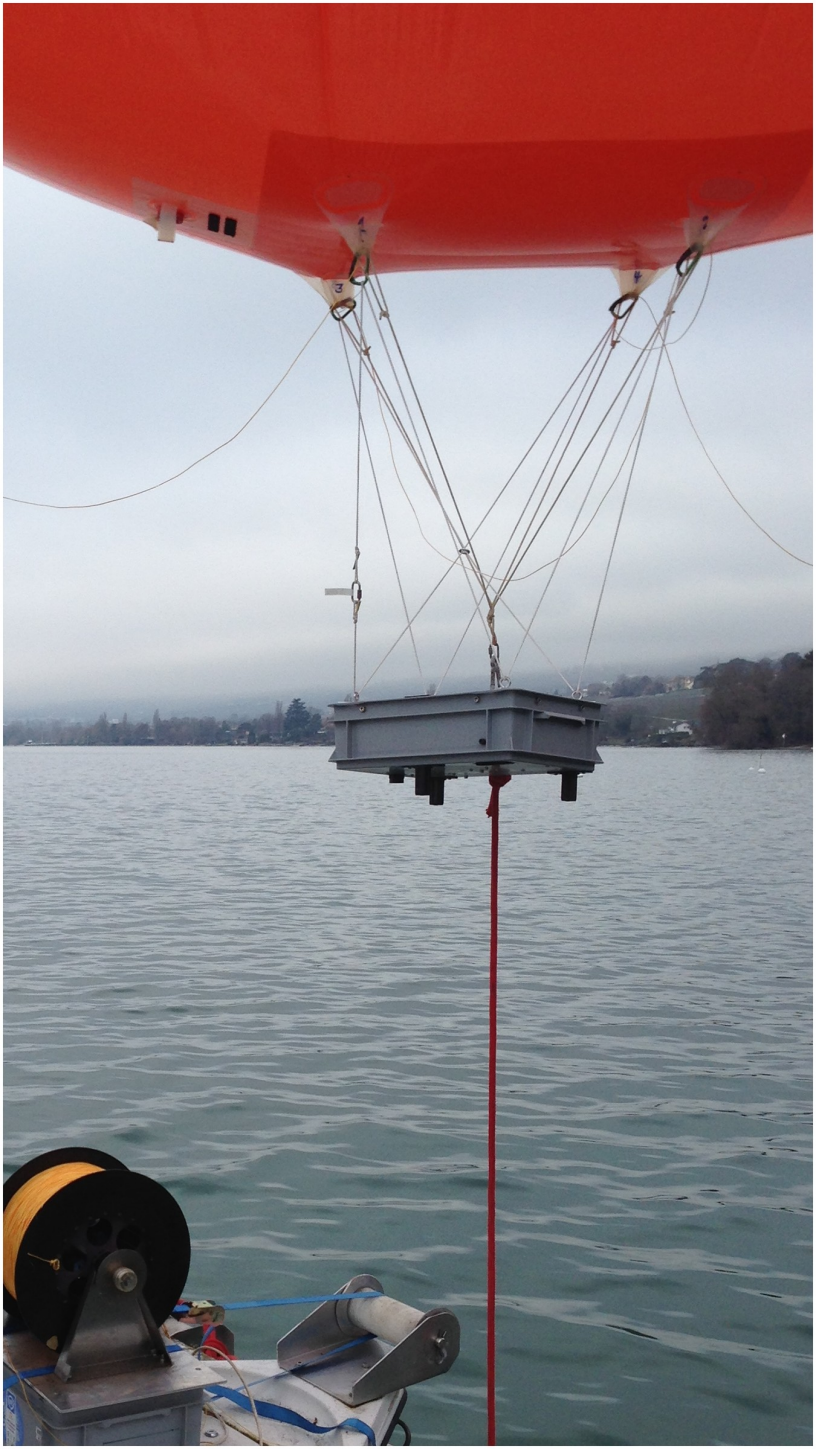
BLIMP system ready for deployment. The balloon is tethered to a winch, which controls its height. It carries the ImPROV instrument package installed in a grey box suspended by a Picavet harness.

**Fig 7:**
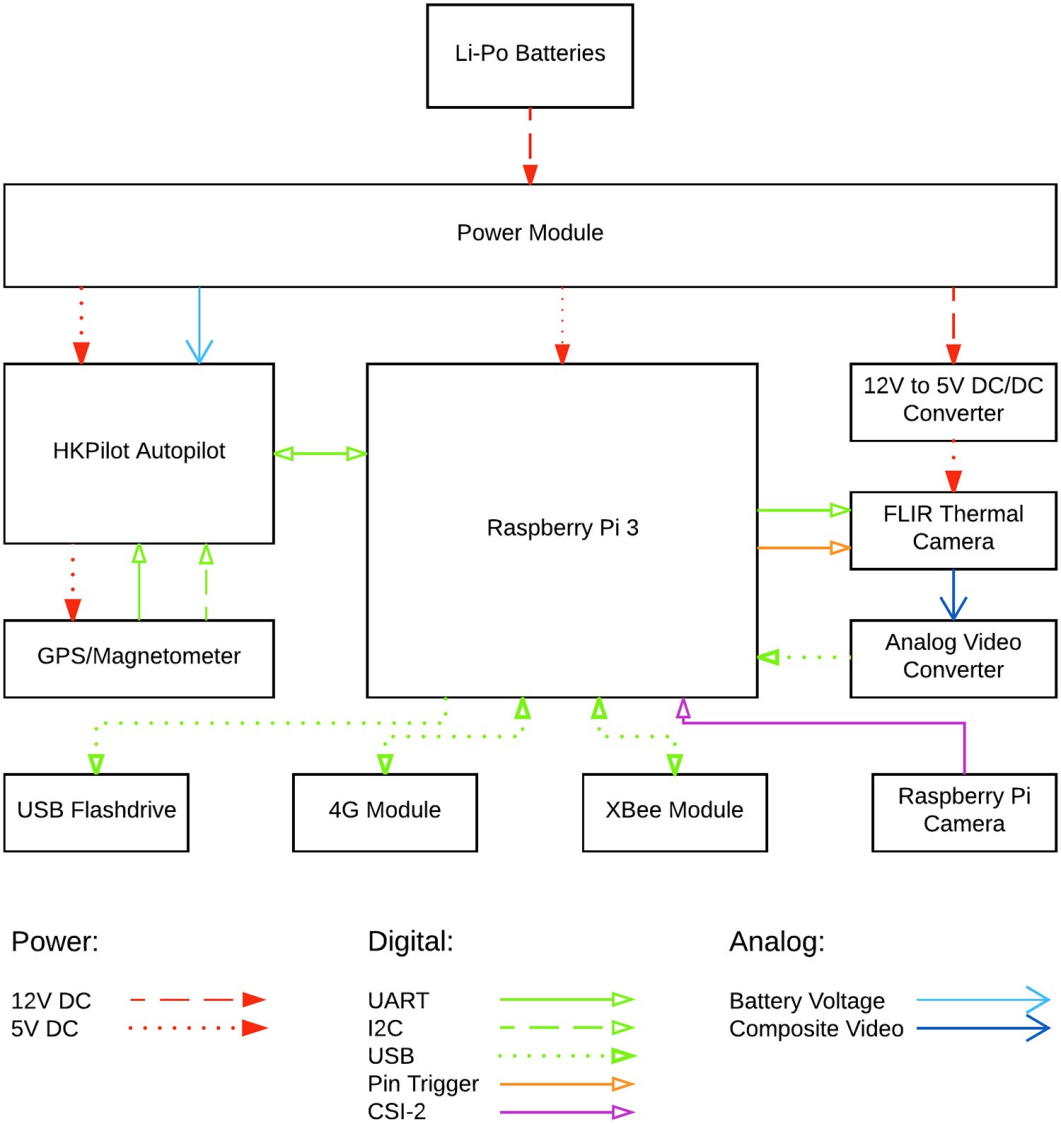
BLIMP imagery package. This schematic shows the different components and their connections in the ImPROV package.

The BLIMP system consists of a 9-m^3^ He-filled balloon that is raised/lowered using a winch (see Fig 6). The BLIMP can reach heights of up to 2 km (however, our missions typically reach less than 600 m elevation). In our deployments, the winch can be tethered to the ZiviCat (for fully autonomous operations) or to the accompanying boat. The BLIMP, which has a payload of about 4 kg, carries a thermal imagery package suspended beneath it. This package, called ImPROV, is depicted in Fig 7. ImPROV is described in detail elsewhere [40], so only an overview is given here.

ImPROV is an adaptable imaging solution for image and video capture (along with location and other metadata), and real-time imagery streaming. In practice, the latter is a valuable feature as missions can be readily changed based on the BLIMP data. For lake surface imagery, ImPROV includes a FLIR Tau 2 LWIR camera and a low-cost RGB Raspberry Pi camera (3280 × 2464 pixels) used for verification purposes. The thermal camera has a resolution of 640 × 512 pixels, includes radiometry capability, and possesses an analog output connected to an analog video converter, which is used for streaming. The thermal camera provides the 14-bit digital outputs (irradiance values as grey levels) that are used for image registration. The thermal image resolution depends on the mission altitude, e.g., 1-m pixel resolution for a flight altitude of ∼475 m (the RGB Raspberry Pi camera gives images with ∼0.15-m resolution at the same altitude). The cameras are controlled by a Raspberry Pi computer, which also manages communication with the base station. The system supports either UDP communication through a mobile network via an LTE module, or serial radio transmission using an XBee Pro 100 mW module. The XBee antenna, with a maximum range of 2 km, is usually used for altitudes where the mobile network signal is weak (in practice above 600 m elevation). ImPROV includes an autopilot for determination of altitude, position and, if needed, attitude. Such metadata are sent to the Raspberry Pi to be recorded with the images. As configured, the thermal camera stores images and associated metadata internally, whereas the corresponding RGB data are stored with images on a separate USB flash drive. Like the catamaran sensor data, in parallel with real-time monitoring, all ImPROV images and metadata are stored on-board for post-mission retrieval.

ImPROV is powered by two 5200 mAh, 3-cell Li-Po batteries running in parallel, giving about 10 h autonomy, which is similar to that for the catamaran. Battery usage is monitored during missions. A power module converts the battery voltage to 5V as required for the autopilot and the Raspberry Pi. The Tau 2 camera possess its own voltage regulator and is thus directly connected to the battery voltage on the power module.

#### BLIMP software

ImPROV includes a Raspberry Pi computer and an autopilot, which communicate while running in parallel. Together, these components handle video recording and streaming, automatic or manual triggers for image capture, metadata recording, and communication with the base station.

The system uses an HKPilot autopilot running the open-source ArduPilot software. Here, the autopilot is simply a convenient means to access to different in-built sensors, viz. IMU, GPS, magnetometer and barometer. The autopilot computes the 6-DOF attitude and position of the system, which are sent to the Raspberry Pi for metadata recording. The Raspberry Pi additionally uses the position data for triggering the cameras for position-based imagery.

Besides controlling the imagery, the Raspberry Pi is responsible for communication/telemetry. The software uses multi-threading to allow simultaneous communication and camera control. The communication thread switches as needed between the XBee radio, the LTE module or the autopilot. Commands are sent to the main thread, which analyzes the command and acts accordingly. For instance, commands are sent that specify the destination camera and the desired camera action such as imaging, recording, streaming, recording/streaming, or turn off. Video recording and streaming are managed by the open-source GStreamer program in a forked thread, allowing for simultaneous RGB and thermal camera viewing at the ground station. For this purpose, camera imagery is compressed using H.264 compression. Fully autonomous image recording (stored losslessly) is achieved using position change or time interval, according to the mission needs.

The BLIMP ground-station communication options—XBee serial radio link or LTE module—offer both redundancy and expand the deployment options. For security, all communications using LTE networks are encrypted.

#### BLIMP base station

**Fig 8:**
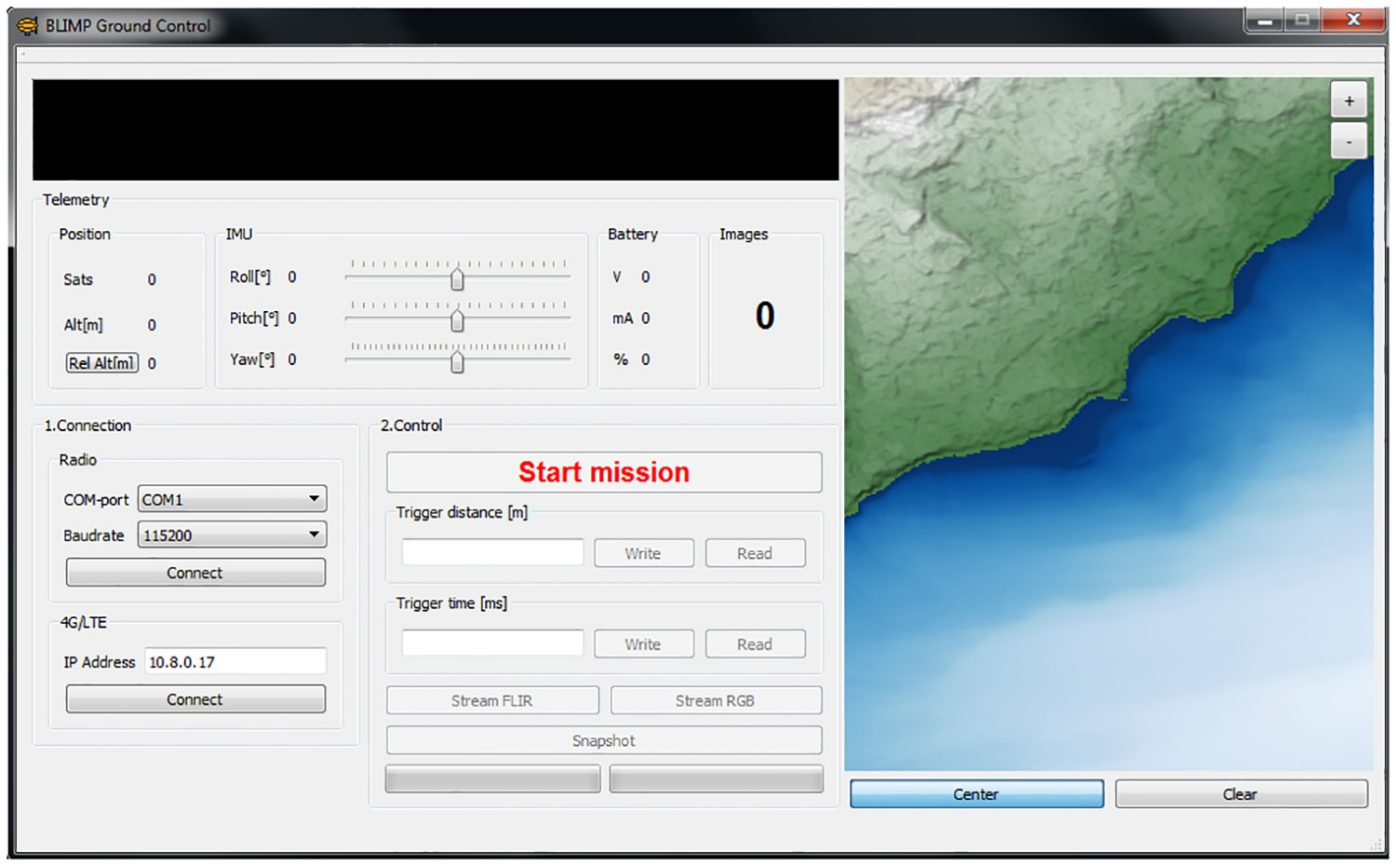
BLIMP base station interface. The ground control interface before launching a mission. Further description is provided in the text. The map in the right panel is for illustrative purposes only, and is adapted from public domain satellite data for topography (NASA SRTM; green area) and lake bathymetry data (SwissTopo; blue area).

The base station for the BLIMP system is used to remotely monitor and control the imagery package, in real-time if desired. It consists of a standard (Windows 7 or later) laptop connected to either a VPN (e.g., through an LTE module) or an XBee antenna. The computer runs a custom user interface that monitors the package and controls the cameras (Fig 8). Telemetry includes roll, pitch, and yaw Euler angles corresponding to the attitude of the package, available satellites for GPS, relative and absolute altitude, battery voltage, and drawn battery current. The user defines the image recording protocol (position or time interval), and streams camera imagery if desired. The system provides a visual indication of real-time position using Open Street Map. Maps are either pre-loaded or accessed via the internet using the LTE link. The actual mission trajectory is tracked as the mission unfolds. The map also displays the estimated frames of the recorded images (using the attitude, altitude and camera FOV). This feature is convenient since the operator can ascertain in real time, for instance, if image overlap (important for subsequent photogrammetry) is sufficient.

## Mission results

Lake surface water temperature (LSWT), which varies spatially and temporally, reflects climatological and meteorological forcing more than any other physical lake parameter. Lake surface and near-surface water temperatures exhibit a direct response to climatic forcing, making epilimnetic temperature a useful indicator of climate change [15]. It has been studied using long-term historical LSWT satellite data (e.g., [18, 41]), in situ measurements (e.g., [42, 43]) and numerical simulations (e.g., [44, 45]). Global warming also increases the duration of thermal stratification, which affects transport and mixing on shorter time-scales.

There are different data sources for LSWT mapping, including remote sensing and in situ measurements. Satellite data, with typical pixel resolution of O(1 km), help identify large-scale thermal patterns, while higher resolution thermography at the sub-pixel scale, i.e., O(1 m) pixel resolution, provides the ability to resolve satellite imagery, and hence to estimate the uncertainty associated with the satellite LSWT values. These thermal patterns also reflect the air-water interactions, mainly surface heat flux and surface shear stress. The surface thermal structures interact with the sub-surface layers through buoyancy, convection and diffusion mechanisms. To capture these processes, simultaneous LSWT mapping and measurement of the near-surface temperature profiles are needed, such as is provided by the BLIMP and ZiviCat.

**Fig 9:**
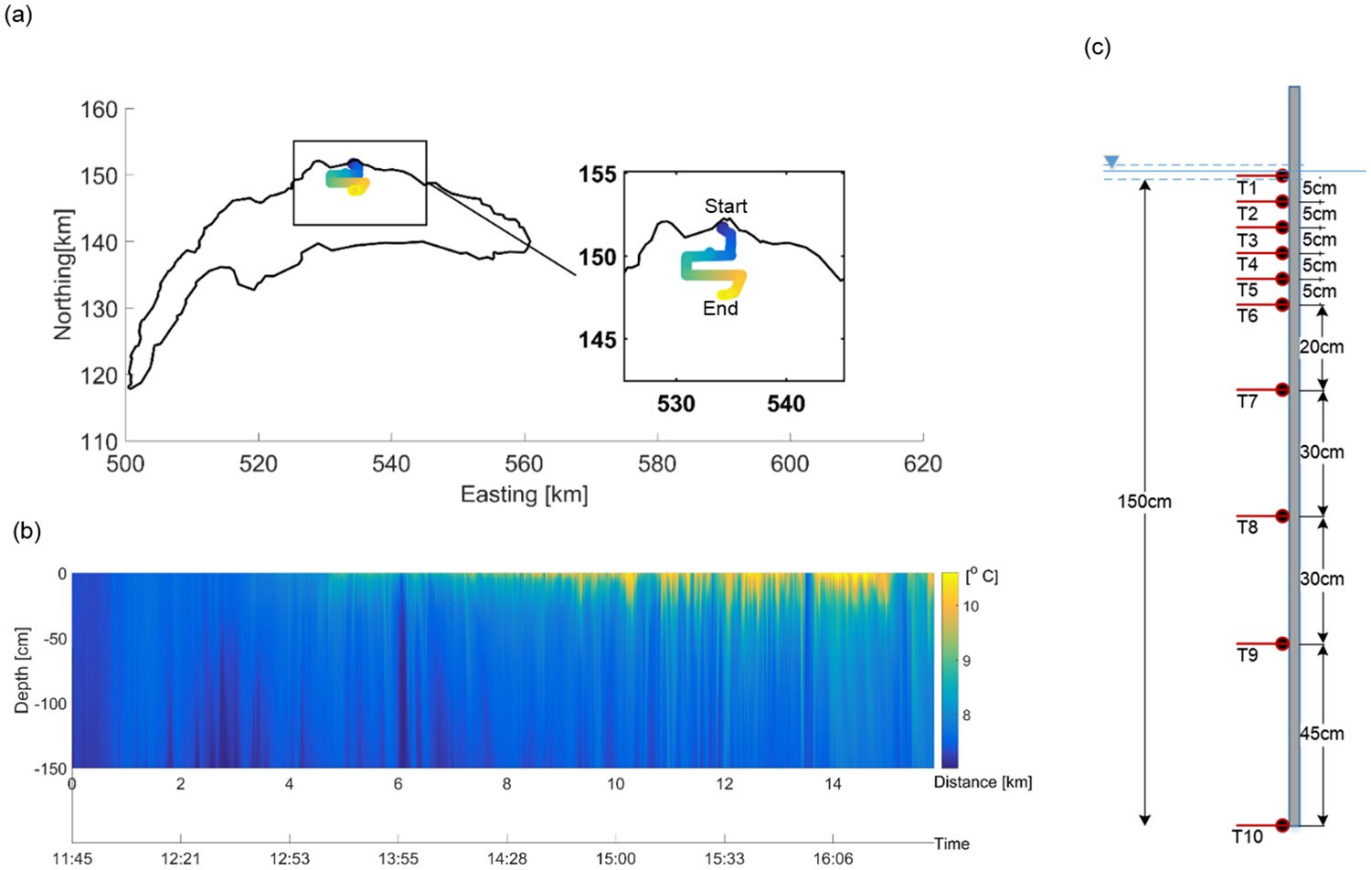
Example of ZiviCat mission results. (a) ZiviCat trajectory on Lake Geneva (colors show temperature from the top-most ZiviCat sensor), 18 March 2016, using the Swiss coordinate system with km length-based units (CH1903); (b) Vertical temperature profiles measured by ZiviCat along the track shown in (a); (c) Thermistor bar configuration.

To show the functionality of the combined ZiviCat-BLIMP system, we present temperature measurements for Lake Geneva from a 5-h field campaign from 18 March 2016. The ZiviCat trajectory (about 16 km) is shown in Fig 9a. The corresponding vertical temperature profiles are presented in Fig 9b. The IMU data track the ZiviCat orientation, and were used to compute precise locations of 10 RBRsolo thermistors in the water column (Fig 9c). As we focused on near-surface processes, more thermistors were located in the upper part of the water column. The vertical temperature profiles demonstrate the development of the near-surface stratified layer as it evolves from an almost fully-mixed condition to one showing buoyant, warmer water in the top 50 cm. This temporal evolution is mainly due to the intense early spring solar radiation.

Some sequential cold/warm fronts are also evident in Fig 9b, particularly during 15:10 to 15:50. Aerial remote sensing is a useful (and probably only) tool to resolve the spatial extent and the direction of such sub-pixel scale features. Previous studies examined sub-pixel scale surface temperature variability using airborne systems. However, due to the challenges intrinsic to thermal image registration over water or instrumental restrictions [39], they only reported along-track point [46] or area-averaged [47] measurements. The developed two-platform measuring system, together with a custom image processing procedure [39], can be used to obtain LSWT patterns at sub-pixel satellite scales.

To obtain the LSWT patterns at sub-pixel scale, the in situ ZiviCat measurements were accompanied by BLIMP imagery, taken on a 5-s interval resulting in more than 90% overlap between sequential images. The thermal images were registered and calibrated using an image processing procedure to create the final LSWT maps with sub-pixel scale resolution. In this procedure, a pixelwise two-point linear correction and a Probability Density Function (PDF) matching in regions of overlap between sequential images were used for non-uniformity (spatial noise) and drift (temporal noise) corrections, respectively. Image stitching was accomplished using feature detection and matching. Features were selected using a combination of the SURF [48] and MSER [49] algorithms. The matching of those points between two frames and their relative geometrical transformation was obtained using MLESAC [50]. A mean value of the overlapped images at each location was considered as a representative value of that pixel in the stitched image. Finally, the measured in situ temperatures were used for the radiometric calibration. It is emphasized that the GPS and temperature data from ZiviCat are essential for ground-truthing the BLIMP images and generating the final LSWT maps. Details of image processing procedure are given in [39].

**Fig 10:**
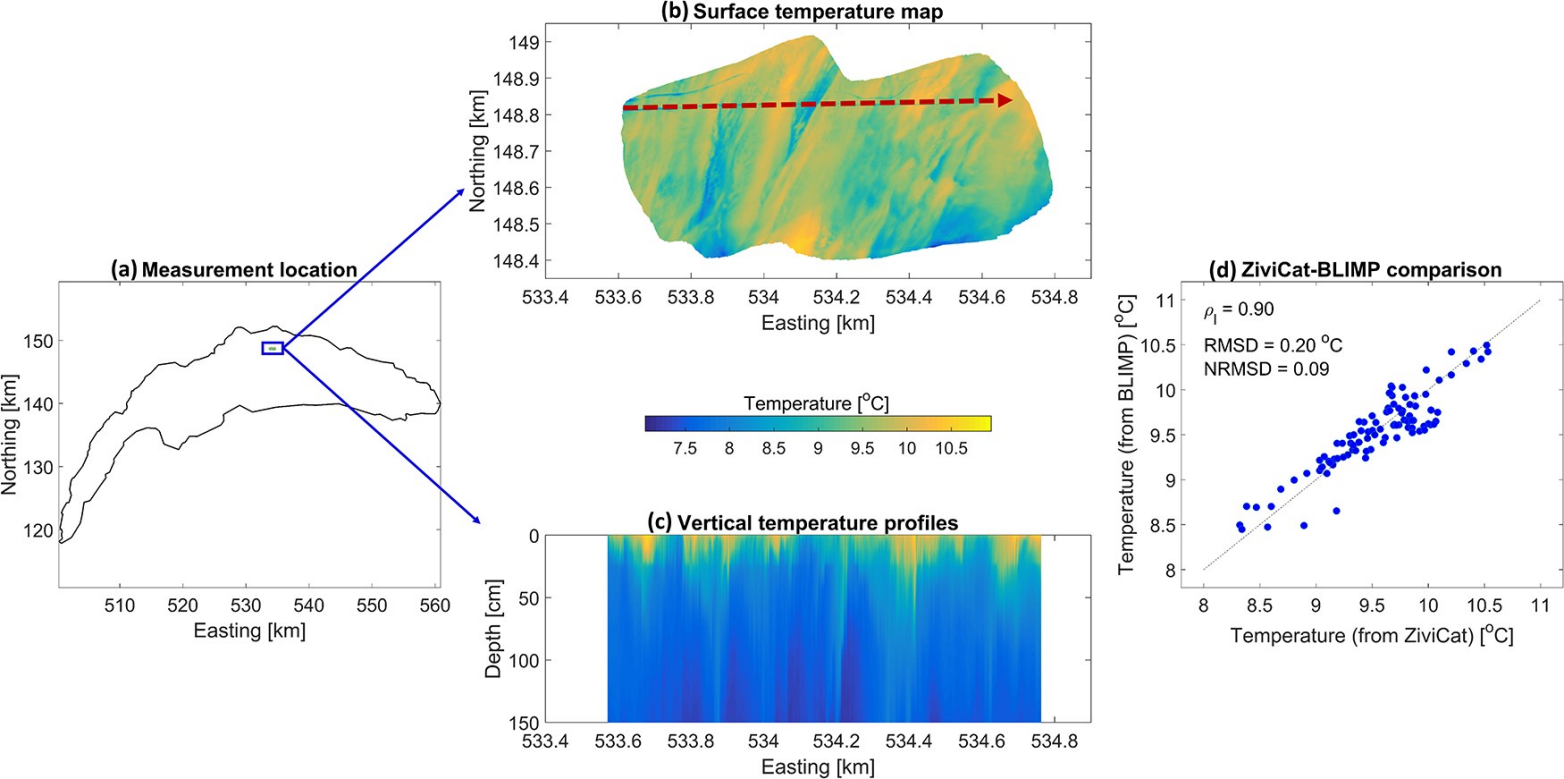
Example of BLIMP mission results. (a) Location of the selected mission on 18 March 2016 at ∼15:30, i.e., the blue rectangle. Axis units use the Swiss coordinate system (CH1903); (b) A stitched composite image of Lake Surface water Temperature (LSWT) with the location on the axes. This map was created using 172 images with a resolution of 0.8 m. A dashed red line marks the track of the ZiviCat and the arrow at the end of the line indicates the direction of motion; (c) Vertical temperature profiles measured along the dashed red line transect using ZiviCat; (d) Comparison of the calibrated LSWT of the stitched image (from the BLIMP) with the corresponding in situ near-surface (2-cm depth) temperatures measured by the ZiviCat. The correlation coefficients for linear curve fitting (*ρ*_*l*_), the Root Mean Square Differences (RMSD) and the normalized RMSD (NRMSD) for the non-linear regression model are given in the legend. The min/max temperature range was used for RMSD normalization.

Fig 10 shows an example composite image (Fig 10b) as well as the corresponding temperature profiles (Fig 10c). The LSWT map (Fig 10b) was created combining 172 images collected over 15 min. The individual thermal images have a spatial resolution of ∼80 cm (BLIMP at ∼380 m above water level). The area covered in Fig 10b, i.e., ∼0.5 km^2^, resolves around half of a typical satellite pixel (1 km^2^). It shows a sequence of horizontal streaks on the lake surface with a temperature range and standard deviation of 2.4°C and 0.3°C, respectively. These streak-like structures are aligned with the wind direction (not shown here). Our on-going research is directed at the relationship between prevailing wind and surface temperature variations, such as those shown here.

Along the catamaran track (indicated by a dashed red line in Fig 10b), some warm/cold temperature variations are observed at the surface. These are in a good agreement with the ZiviCat temperature profiles (Fig 10c). The comparison between the near-surface (2-cm depth) measured temperatures (from ZiviCat) and the calibrated LSWT values (processed BLIMP data) from the thermal maps of Fig 10b are shown in Fig 10d. The results indicate a correlation coefficient of 90% and a Root Mean Square Difference (RMSD) of 0.2°C between near-surface ZiviCat temperatures and the calibrated BLIMP values. The observed deviations between ZiviCat and BLIMP LSWTs can be due to errors in the estimation of the geometric transformations, the difference between skin (top 10–500 μm layer) and near-surface temperatures (e.g., [51]), uncertainties in the ZiviCat measured and corrected data, and errors associated with denoising the BLIMP images and their radiometric calibration [39].

This combined surface-aerial lake measurement platform can be used to study physical, chemical and biological processes. For example, systematic measurements over larger areas over wider range of conditions, and possibly at different sites, can be used for satellite ground-truthing [47], and can provide better insight into spatial heterogeneity of LSWT warming rates, within [52] and among lakes [53]. The measurements can also improve understanding/quantification of mass, heat and momentum exchanges at the air-water interface (e.g., [54, 55]) and so improve numerical weather prediction results (e.g., [56, 57]). Visualizing LSWT details using this platform can also be achieved for river inflows, wastewater discharges and near-shore processes, e.g., thermal biomes, all of which will affect the lake ecosystem dynamics.

## Conclusion

ZiviCat is a modular, low cost, autonomous mobile platform. The low cost is ensured by use of a commercial catamaran hull, which was straightforwardly modified as a stable, autonomous platform and permitted rapid conversion. A second major design choice was the use of readily available components. Of course, this approach cannot avoid the challenge of system integration and the development of software and hardware necessary as part of any custom-built craft. A mechanical workshop was needed to convert the catamaran hull. The power and navigation systems used common components as much as possible. Other parts were made as needed, e.g., using a consumer grade 3D printer to make the PCB rack described in Part A of S1 Text. Sensor integration must be done on a case-by-case basis, but many sensors use standard protocols such as USB or SPI, which work unmodified with our present system. In general, however, some expertise in electronics is needed to build a replica of the ZiviCat system, in addition to knowledge of the C++ language. The in situ and trajectory-based measurements of the ZiviCat are complemented by lake surface imagery obtained with the BLIMP, which can operate as an independent platform if so desired. The BLIMP contains the ImPROV system, an adaptable imaging solution that features full remote control, redundant communication and different options for camera imaging.

## Supporting information

S1 Text Details of platforms components. (PDF)
