# Supplementary material for: A low-cost, autonomous mobile platform for limnological investigations, supported by high-resolution mesoscale airborne imagery
Source: PLoS One. 2019 Feb 14;14(2):e0210562. doi: 10.1371/journal.pone.0210562 (PMC6375554; doi:10.1371/journal.pone.0210562)
Supplement: S1 Text — (PDF) [file pone.0210562.s001.pdf]

A Low-Cost, Autonomous Mobile Platform for Limnological Investigations, Supported by High-Resolution Mesoscale Airborne Imagery

Supporting Information

D. Andrew Barry[1,*], Jean-Luc Liardon[1], Philippe Paccaud[1], Pascal Klaus[1], Nawaaz S. Gujja Shaik[1], Abolfazl Irani Rahaghi[1], Ludovic Zulliger[1], Jérôme Béguin[1], Beat Geissmann[1], Stepan Tulyakov[2], Anton Ivanov[2], Htet Kyi Wynn[1], Ulrich Lemmin[1]

[1] Laboratoire de technologie écologique (ECOL), Institut d'ingénierie de l'environnement (IIE), Faculté de l'environnement naturel, architectural et construit (ENAC), Station 2, Ecole polytechnique fédérale de Lausanne (EPFL), 1015 Lausanne, Switzerland

[2] Space Engineering Center (ESC), Station 13, Ecole polytechnique fédérale de Lausanne (EPFL), 1015 Lausanne, Switzerland

[*]andrew.barry@epfl.ch

Part A. ZiviCat Components. This list describes the major components of the ZiviCat system. The different components are shown in Fig 2.

- **H1 - Hull:** For the platform of the ZiviCat system, we chose the dual-hull, 5.18-m long, 2.41-m wide Hobie Cat 17 frame. It is modified to retain only the hulls and the joining crossbars.
- **H2 - Beams:** Aluminum beams, mounted on the hulls, are used to support the system and sensors.
- **H3 - Decks:** On the aluminum beams, four water-resistant plywood boards are set up to hold the system components.
- **P1 - Low-noise gasoline generator:** This powers the entire platform, supplying 230V at 50 Hz at a maximum power rating of 2 kW. A full (6-L) tank runs the entire system for approximately 8 h. The generator was selected as it delivers a stable sinusoidal power signal and is not prone to voltage drops that could disrupt some elements within the logic box, mainly the central computer. Link: `https://www.yamahamotorsports.com/generator/models/inverter-ef2400ishc` (last accessed 17 November 2018).
- **P2 - Power Box**
  - **I - 230V AC to 24V/12V/5V DC converter:** This converter is dedicated to supplying power to the Logic Box at different voltages for the different electronic components and sensors of the Logic System.
  - **II - 230V AC to 12V DC converter:** This converter supplies power to the motors through the motor drive circuitry. The propulsion power system is electrically isolated from the logic system to prevent any potential crossover power issues.
  - **III - 12V Lead-acid battery:** Connected in parallel to the P2.III converter, this battery acts in similar manner to a capacitor to protect the circuitry from current spikes, which can occur either on motor power-up, or during operation when the PWM power signal switches to the ON state. Without this battery, the surge current within the motor will pull down the voltage rail of the P2.III converter to a low level, causing it to stop supplying power by entering a safety mode.
  - **IV - Distribution Box:** Used to distribute the 230V AC signal from the P1 generator to the converters. At system power-up, all of the current supplied from the generator flows through two separate 150-Ω, 100 W-rated resistors, one for each converter. After 1 s, a timer switches on a bypass circuit and the current from the generator flows directly to the converter. This allows the system to reduce the strain on the generator due to inrush currents and stabilizes the start-up before entering the operations mode.
  - **V - Motor Drive Circuitry:** Consists of a Sabertooth motor drive that outputs the proper PWM power signals to the motors based on a serial input command. The PWM signal modulates the voltage level that is fed into the motors. As the propeller speed is proportional to the voltage, changing the duty cycle of the PWM signal permits control of the propeller speed through voltage changes. The motor drive is capable of using regenerative braking and restoring inductive energy within the motor in order to maximize efficiency. Link:

https://www.dimensionengineering.com/products/sabertooth2x25 (last accessed 17 November 2018).

- **P3 - Motors:** Two Minn Kota Traxxis mounts using brushed DC propeller motors. These two motors provide the entire propulsion to the system. At a maximum thrust of approximately 245 N, supplied by 12V, they allow the entire platform to move at about 1 m/s at full load. The ZiviCat's speed is about 2 m/s when unloaded. Link: https://www.minnkotamotors.com/support/manuals/trolling-motors/traxxis (last accessed 17 November 2018).

- **P4 - Winch:** The winch is used to immerse a CTD probe in the water. It is controlled by the L1.II BBB using a MAX3232 chip, allowing for conversion from 3.3V logic levels to required RS-232 signals. The 230V power supply comes directly from the generator.

- **L1 - Logic Box**

  - **I - Distribution Board:** The 5V and 12V voltages from the P2.II converter go through this board in order to be rerouted to the different components within the logic subsystem based on their need. Several unused slots are available for use by future sensors or components within the logic system. Furthermore, this board is also used to reroute motor instructions from the L1.III Arduino to the P2.VI Motor drive circuitry.
  - **II – Beaglebone Black (BBB) + Shield:** The central processing on-board computer of the entire platform. It handles navigation, sensor data accumulation, processing and saving, and communication with the base station using the L3 XBee Antenna (or L2 Wi-Fi Antenna). All system and sensor data are stored on an on-board SD card. The BBB also streams all the available sensor data information to the base station in real time, so that the operator can modify mission parameters on-the-fly if necessary. A custom shield extension PCB is used to facilitate connections with peripherals and to the L1.III Arduino, the L1.IV SPI rack and the L4 GPS antenna. Link: https://beagleboard.org/black (last accessed 17 November 2018).
  - **III - Arduino Leonardo + Shield:** Arduino is an open-source, easy-to-use microcontroller board that serves as a secondary on-board processing unit. Its sole purpose is to ensure that the autonomous navigation mode can at any moment be overridden by manual controls, including in cases where the primary L1.II on-board computer is non-responsive or offline. The Arduino controls a multiplexer that reroutes the motor commands either from the L1.II BBB (in automatic mode) or from the Arduino itself in manual mode. The remote control signals from the operator are received through the connected L1.VII RC antenna in the form of a PWM signal, which also serves as the switching signal for the multiplexer. Signals are then converted into motor commands and transmitted to the P2.VI Motor Drive Circuitry. Link: https://www.arduino.cc/en/Main/ArduinoBoardLeonardo (last accessed 17 November 2018).
  - **IV - SPI Rack:** A custom-made PCB rack designed to accommodate up to 15 sensor interfacing PCBs. Every board within this rack is designed to interface with one sensor type (multiple sensors of the same type can be interfaced through a single board). Usually, the board consists of analogue-to-digital converters with an SPI interface, along with

sensor-specific microchips, and in some cases can also be used as a voltage source. The rack itself contains a multiplexer that allows the L1.II BBB to read measurements of all the interfaced sensors using a single SPI line.

  - **V - USB hub:** This hub allows sensors or peripherals that use a USB connection to interface with the L1.II BBB.
  - **VI - Attitude and heading reference system (AHRS):** A sensor package consisting of 3-axis MEMS accelerometers and gyroscopes along with a magnetometer and an in-built processor. Unlike IMUs that simply output sensor data, the AHRS computes and outputs attitude and heading data as well. Combined with GPS data, this system is used for the localization and navigation of the ZiviCat platform.
  - **VII - RC antenna:** Allows the L1.III Arduino to receive RC commands from an operator in manual mode, which overrides the automatic navigation of the platform. Field testing shows that the RC link has a range of approximately 400 m.

- **L2 - Wi-Fi antenna:** At system boot, the L1.II BBB creates a local Wi-Fi hotspot and network. It is especially useful for troubleshooting or debugging the system since it allows an operator to quickly interface with the L1.II BBB using an SSH link. In open waters, the effective range is about 200 m with a bandwidth of up to 100 Mbits/s.

- **L3 - XBee antenna:** A two-way 2.4 GHz radio communications antenna. It is the main peripheral through which the system status and sensor data are streamed to the base station. It provides a reliable connection of up to 250 kbits/s at a range of up to 3 km. Both this antenna and the L2 Wi-Fi antenna are installed at the top of the main mast. Link: `https://www.digi.com/lp/xbee` (last accessed 17 November 2018).

- **L4 - GPS antenna:** Standard GPS antenna used to obtain geolocation information for the platform.

- **L5 - Sensors:** The currently installed sensors are listed below:

  - **I - Temperature sensors (10×):** The RBRsolo temperature sensors have an accuracy of 0.002°C and communicate with the L1.II BBB through a RS-232 communication standard. Within the L1 Logic Box, there are RS-232-to-USB converters that allow the sensors to be interfaced through the L1.V hub. The sensors themselves are installed at multiple positions on a vertical metal bar immersed in the near-surface region, giving temperature profiles of the upper 1.5 m of the water column.
  - **II - Pressure sensor:** This sensor is attached to the same metal bar as the L5.I temperature sensors, at the deepest position. The pressure data are delivered as an analogue signal that is converted to digital by the dedicated sensor board in the L1.IV rack. The reference pressure for the sensor is taken within the L1 Logic box.
  - **III - Echologger:** Echologger EU400. Used for measuring the depth at any given position, it has a range of up to 100 m. This sensor is interfaced with the L1.II BBB using a USB connection and the L1.V hub. It is mounted at the end of the immersed bar.
  - **IV - Humidity sensor:** Installed on the flag pole. A standard ADC converter board installed in the L1.IV SPI rack converts the analogue output signal of the sensor to usable digital format.

- V - Anemometers: Two anemometers can be simultaneously mounted on the platform. They are connected to a board on the SPI rack that takes as input the measured rotating frequency and potentiometer values and transforms them into wind speed and wind direction, respectively.
- **VI - Radiometer:** CNR 4 Net Radiometer with upwards and downwards facing pyranometers and pyrgeometers, along with an internal PT100 temperature sensor. The radiometer is fixed at the tip of a deployable horizontal bar that, when in position, puts the sensor well beyond the front end of the platform. This is done in order to have measurements without the interference due to the wake of ZiviCat when it is in motion. The PT100 temperature sensor, pyranometer and pyrgeometer analogue data signals are converted to digital using dedicated boards installed in the L1.IV rack.
- **VII - Acoustic Doppler Current Profilers (ADCPs):** This instrument measures water velocity profiles along a water column underneath it, using multiple sonars. One is used pointing upward to measure the water flow at the surface, and one is pointing downward to measure the flow underneath the platform.
- **VIII - CTD Probe:** The CTD90M from Sea & Sun Technologies is immersed in the water to record conductivity, temperature, and the depth at which the measurements are taken. It is mounted on the winch for automatic measurements at defined locations. It uses an FSK protocol allowing for several hundred meters' cable length. With the probe comes an FSK-to-RS-232 converter, which is further converted to USB and connected to the L1.V USB Hub.

- **L6 - Obstacle detection system:** This system is a modular add-on to the ZiviCat platform [1]. It carries out image processing tasks, facilitated with a dedicated Raspberry Pi 3 single-board computer, with images obtained with a Raspberry Pi camera. The system communicates with the L1.II BBB through a local wireless network, giving options of mission modification and/or operator notification, depending on the mission type.

Part B. BLIMP Components.

- **Li-Po Batteries (2×):** The system is powered by two 5200 mAh 3S batteries connected in parallel, giving a mission duration of up to about 10 h.
- **Power module:** This module is comprised of a 12V-to-5V DC/DC converter that supplies the power to both the autopilot and the Raspberry Pi. It also measures the voltages and current supplied from the batteries and transmits this information to the autopilot.
- **DC/DC converter:** The cable connecting the FLIR thermal camera to the power source has an in-built DC/DC converter. As such, the camera is directly connected to the battery system instead of being supplied by the power module.
- **Tau 2 FLIR Thermal Camera:** The camera system includes a Teax frame grabber along with an internal SD card for storing images. It is triggered by the Raspberry Pi, with full digital images and associated metadata (GPS, IMU, time) stored internally. Analogue video is used for streaming images to the base station. Links: `http://www.flir.com/cores/display/?id=54717` (last accessed 17 November 2018), `http://www.teax-tec.de/index.php?id=23&L=1` (last accessed 17 November 2018).

- **HKpilot Autopilot:** This autopilot runs a slightly modified version of the open-source ArduPilot software, and is used for its sensors, sensor interfacing, and as a secondary processing unit. It computes the attitude of the BLIMP using an easily interfaced GPS/magnetometer module and its internal IMU. The resulting estimate is transmitted to the Raspberry Pi. The autopilot also monitors the battery state. When the system is configured to capture images based on distance increments, the autopilot computes the travelled distance based on its GPS information and sends the image capture command to the Raspberry Pi. Link: `https://hobbyking.com/en_us/hkpilot32-autonomous-vehicle-32bit-control-set-w-power-module.html?___store=en_us` (last accessed 17 November 2018).

- **Raspberry Pi 3:** The Raspberry Pi 3 single-board computer is used as the central processing unit of the BLIMP system. It handles all of the essential processes of the platform such as communication, camera control and management, as well as data storage. It controls the RGB camera and triggering of the thermal camera, while sending associated image metadata. It defines the current system state (imaging, streaming, etc.) and communicates mission data to the base station while receiving and rerouting any instructions to the relevant system parts. Link: `https://www.raspberrypi.org/products/raspberry-pi-3-model-b/` (last accessed 17 November 2018).

- **Analog Video Converter:** The video stream from the FLIR cannot be directly processed on the Raspberry Pi in its analogue form. The converter transforms the signal from analogue to a USB serial data stream. In fact, this device allows the Raspberry Pi to receive the video stream as it would from a standard webcam device.

- **Raspberry Pi Camera V2:** This 8-MP camera captures RGB images during missions. The image capture is synchronized with the thermal images from the FLIR camera. The Pi camera is interfaced to the Raspberry Pi computer using the dedicated interface. This, along with the in-built camera commands results in a versatile and easily manageable system. During a mission, the camera can simultaneously stream 640×480 video to the ground station using H.264 compression. Link: `https://www.raspberrypi.org/products/camera-module-v2/` (last accessed 17 November 2018).

- **GPS/Magnetometer Module:** Module containing a GPS antenna for geolocation information and a magnetometer used to complement the internal IMU unit of the autopilot to obtain information on the platform orientation.

- **USB Flash drive:** Standard USB storage drive used to save the RGB images along with the mission log.

- **4G Dongle:** The 4G LTE USB dongle enables the system to communicate with the base station through the internet, and allows for video streaming. Depending on signal coverage, the system can communicate with the base station up to a relative altitude of 600 m, above which the XBee radio antenna is used.

- **Radio antenna:** At balloon elevations above the reach of the 4G coverage, the radio antenna is used to establish a link between the system and base station to allow for continued monitoring. It can also be used to transmit images, but at a low rate (about 13 images/min).

Part C. Wireless Internet Communications with 3G/4G.

Here, we describe an easy-to-implement method which transforms 3G/4G mobile networks into reliable, high-bandwidth communication channels between multiple users and devices, any or all of which are independent of a wired network (S1 Fig). It relies on a computer server (hereafter "server") that communicates between users and devices (each user and device hosts a computer). Multiple users and devices can operate seamlessly with the presented method. The server is the key to unlocking communications between users and devices, which are otherwise blocked by mobile network providers. Specifically, operators block UDP (User Datagram Protocol) connections and use NAT (Network Address Translation), with the upshot that 3G/4G connections (users or devices) on the 3G/4G network do not have remotely accessible IP (Internet Protocol) addresses.

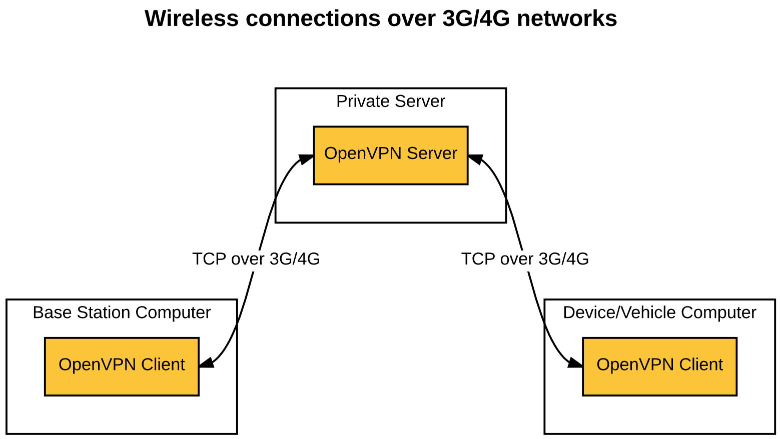

S1 Fig. Schematic of the OpenVPN communication solution presented here.

Communication in the absence of public IP addresses is impossible. This limitation is bypassed by creating a VPN (Virtual Private Network). There are different kinds of VPN, the main ones being:

- L2TP (Layer 2 Tunneling Protocol)
- IPSec (Internet Protocol Security)
- PPTP (Point-to-Point Tunneling Protocol)
- OpenVPN (Open source VPN)

OpenVPN (last accessed 17 November 2018) was selected for several reasons:

- Open source, free and well documented
- Strong internal security
- Easy setup
- Highly configurable
- Works on any port (unlike other protocols)
- Versatile, since it is implemented on numerous operating systems

Details of the method differ for the server and remote devices (OpenVPN is installed on all computers).

Server

The server has a fixed (or, at least, known) IP address, i.e., can be addressed by the clients. A dynamic DNS (Domain Name System) service can be employed if the IP address is not fixed. In addition, the server has an open and free port (also needed for remote access). If there are no open ports (because of gateway limitations), then port 80 (internet) can be used, although this means that the server cannot simultaneously host a website.

There are two main things to setup on the server: (i) keys and certificates, and (ii) the server configuration file.

Keys and certificates

Certificates and keys for the OpenVPN server, and subsequently for clients (i.e., users and devices) connecting to the server, are created using a private Certificate Authority (CA). Following the OpenVPN documentation, the steps are:

1. Generate certificate and key for the server
2. Generate certificates and keys for user and device computers

Server configuration

The server can be configured using only one file. The default configuration file provided with OpenVPN is modified as follows:

- **port 1194:** Define the port used by OpenVPN. Port 1194 (the default) can be replaced by any unused open port. As noted already, port 80 is used if all other ports are blocked by a firewall.
- **proto tcp:** Choose proto tcp over proto udp, i.e., the TCP (Transmission Control Protocol) protocol is used over UDP.
- **ca, cert and key:** These are the certificate and key files that were generated previously.
- **client-config-dir ccd:** Here, ccd is a directory (within the default OpenVPN directory) where the clients are identified. Each client has its own profile containing a single line of text specifying a fixed IP address for the client (i.e., user or device). Thus, for each client, create an IP address file in the ccd folder using a name identifying the client. In the file, put "ifconfig-push 10.8.0.X 10.8.0.Y", where Y = X + 1. The first number is client's IP address and the second the virtual server IP that it connect to (used only by the client). Note that this setup permits one client to communicate with another having a known IP address on the VPN server, i.e. insofar as the clients are concerned, the different "10.8.0.X" addresses are used.
- **client-to-client:** Allow different clients to "see" each other.

Clients (user and device computers)

A configuration file and the previously generated keys and certificates are added to the clients (which, like the server, are running OpenVPN). For each client, a new configuration file is created by copying the same default client file as used on the server. The following fields are then modified:

- **proto tcp:** Choose proto tcp over proto udp.

- **remote my-server-1 1194:** my-server-1 is the server IP address and 1194 is the previously specified server port.
- **ca, cert and key:** These are the files generated previously for the client.

The client's .ca, .cert and .key files are located in the same directory as the configuration file.

References

1. Paccaud P, Barry DA. Obstacle detection for lake-deployed autonomous surface vehicles using RGB imagery. PLOS ONE. 2018;13(10):e0205319. doi:10.1371/journal.pone.0205319.
